# The complete chloroplast genome of *Camellia brevistyla* (Hayata) Coh. St. (Theaceae: Ericales) from China based on PacBio and Illumina data

**DOI:** 10.1080/23802359.2021.1917320

**Published:** 2021-07-14

**Authors:** Xin Yin, Bin Huang, Bo Wang, Li-an Xu, Qiang Wen

**Affiliations:** a Jiangxi Provincial Key Laboratory of Camellia Germplasm Conservation and Utilization, Jiangxi Academy of Forestry, Nanchang, China; b Co-Innovation Center for Sustainable Forestry in Southern China, Nanjing Forestry University, Nanjing, China

**Keywords:** *Camellia brevistyla*, chloroplast, PacBio sequencing, phylogeny

## Abstract

*Camellia brevistyla* is an economic plant that can produce high-value edible oil in southern China. Using a combination of PacBio RS and Illumina sequencing platforms, the complete chloroplast genome of *C. brevistyla* was assembled and annotated. This newly deciphered chloroplast genome was 2,731 bp shorter in the *ycf1* gene than the previously published *C. brevistyla* genome. The phylogenetic analysis fully resolved *C. brevistyla* in a clade with *C. kissii*, *C. chkeiangoleosa*, and *C. japonica.* The results not only supported the proposal to merge the sections *Oleifera* and *Paracamellia,* but also showed the close relationship between them and section *Camellia*.

*Camellia brevistyla* is a valuable woody edible oil germplasm resource and model species (2*n* = 30) classified to section *Paracamellia* in the genus *Camellia* (Chang 1981). The seed oil of *C. brevistyla* has anti-inflammatory, anti-oxidant, and regulating effects on intestinal inflammations (Wang et al. 2019; Wu et al. 2020), and its seed pomace was also shown to be effective in treating hypertension (Chiang et al. 2019). The phenotypic characteristics and reproductive stage of *C. brevistyla* are consistent with that of *Camellia oleifera*, while the former has smaller flowers, fruits, and leaves, but higher oil content. Chang (1981) divided sect. *Paracamellia* into two sections, sect. *Oleifera* and sect. *Paracamellia*. However, due to the similar phenotypic characteristics, Ming and Zhang (1996) merged sect. *Oleifera* into sect. *Paracamellia*. In view of the controversy of the phylogenetic relationship between two sections (Chang and Ren 1998; Ming 2000), obtaining more chloroplast genomes for molecular phylogenetic analysis is particularly important. Wang et al. (2020) presently obtained the chloroplast genome of *C. brevistyla* by using Illumina sequencing. However, we observed that its length was different from the published chloroplast genomes of 48 other *Camellia* species. Using Pacbio RS along with Illumina sequencing, we obtained the complete chloroplast genome of *C. brevistyla* from Jiangxi, China to test the length polymorphism, contribute to further studies on the evolutionary phylogeny of this taxon, and for the conservation efforts of *C. brevistyla*.

The samples of *C. brevistyla* were collected from Wuyi Mountain (Jiangxi, China; coordinates: 27°49′59.88′′N, 117°44′28.98′′E; altitude: 1485 m), and were reserved in the Key Laboratory of *Camellia* Germplasm Conservation and Utilization, Jiangxi Academy of Forestry (specimen voucher: DZC036). Total genomic DNA was extracted from fresh leaves using TRIzol Reagent (Invitrogen, California, USA). For the Pacific Biosciences sequencing, 20k insert whole-genome shotgun libraries were generated and sequenced on a Pacific Biosciences RS instrument using standard methods. First, we used ABySS (http://www.bcgsc.ca/platform/bioinfo/software/abyss) to perform the genome assembly with multiple-Kmer parameters and to identify the optimal settings of the assembly. Secondly, canu v2.1.1 (https://github.com/marbl/canu) was then used to assemble the corrected Pacbio long reads. Finally, GapCloser software (https://sourceforge.net/projects/soapdenovo2/files/GapCloser/) was subsequently applied to fill up the remaining local inner gaps and correct the single base polymorphisms for the final assembly results. A total of 4.70 G clean data (Q20: 98.5%) and 61,692 subreads (N50: 4735 bp) were obtained by Illumina sequencing and Pacbio RS (SRX10153405), respectively. All gene models were performed using the blastp against the non-redundant database (NR in NCBI) and SwissProt (http://uniprot.org).

The chloroplast genome of *C. brevistyla* was uploaded to GenBank (Accession Number: MW256435). It is similar in length and organization to other *Camellia* species published in the NCBI database. The chloroplast genome of *C. brevistyla* was 156,550 bp in length with 37.53% GC content, including a pair of inverted repeat (IR) regions (25,947 bp each), a large single-copy region (LSC; 86,264 bp), and a small single-copy region (SSC; 18,392 bp) regions. A total of 107 unique genes was annotated, including 79 unique protein-coding genes, 24 tRNA, and four rRNA, which are similar to those in other *Camellia* genomes (Yang et al. 2013). The length of the chloroplast genome of *C. brevistyla* from Jiangxi was 2731 bp shorter in length than that published by Wang et al. (2020). The difference mainly appeared in the *ycf1* gene at the boundary between the SSC and IRa. The length of the *ycf1* gene in our assembly was only 5620 bp, while it was 8356 bp in Wang’s study. It is suspected that this difference was caused by assembly errors due to the assembly and sequencing methods. Here we found the same reverse sequence from *rpl32* to the boundary of IRa/SSC, including the whole coding sequence of the *ndhF* gene.

The phylogenetic analysis was performed based on the alignment of complete chloroplast genomes of 24 published *Camellia* species. A bayesian-inference (BI) phylogenetic tree was reconstructed using MAFFT v7.475 and Mrbayes v3.2.6 with nucleotide substitution model GTR + I + G (Huelsenbeck and Ronquist 2001; Katoh and Standley 2013), and the tree was visualized using Figtree v1.4.3. Two species from the genus *Symplocos* (*S. ovatilobata* and *S. costaricana*) were used as an outgroup. As shown in Figure 1, *Camellia* species have a close relationship with 100% posterior probability, and *C. brevistyla* was closely related to *C.kissii*. Although there was a significant difference from Wang et al. (2020) in the gene *ycf1*, a continuous 2736 bp sequence fragment, it did not affect the phylogenetic position of *C. brevistyla*. The phylogenetic tree constructed in this study was in general consistent with the classification of *Camellia* written by Chang (1981). Obviously, the phylogenetic tree inferred using the whole chloroplast genome sequence had a higher resolution than those using common various barcodes (e.g. *matK, rbcL, trnL-F*, *rps16*, etc.) (Zhang et al. 2014). The difference between this phylogenetic hypothesis and that reported by Wang et al. (2020), may be due to the different data sets and different models used for phylogenetic analysis. In addition, the phylogenetic tree also revealed that species from sect. *Paracamellia*, sect. *Oleifera* and sect. *Camellia* are closer in relationship.

**Figure 1.**
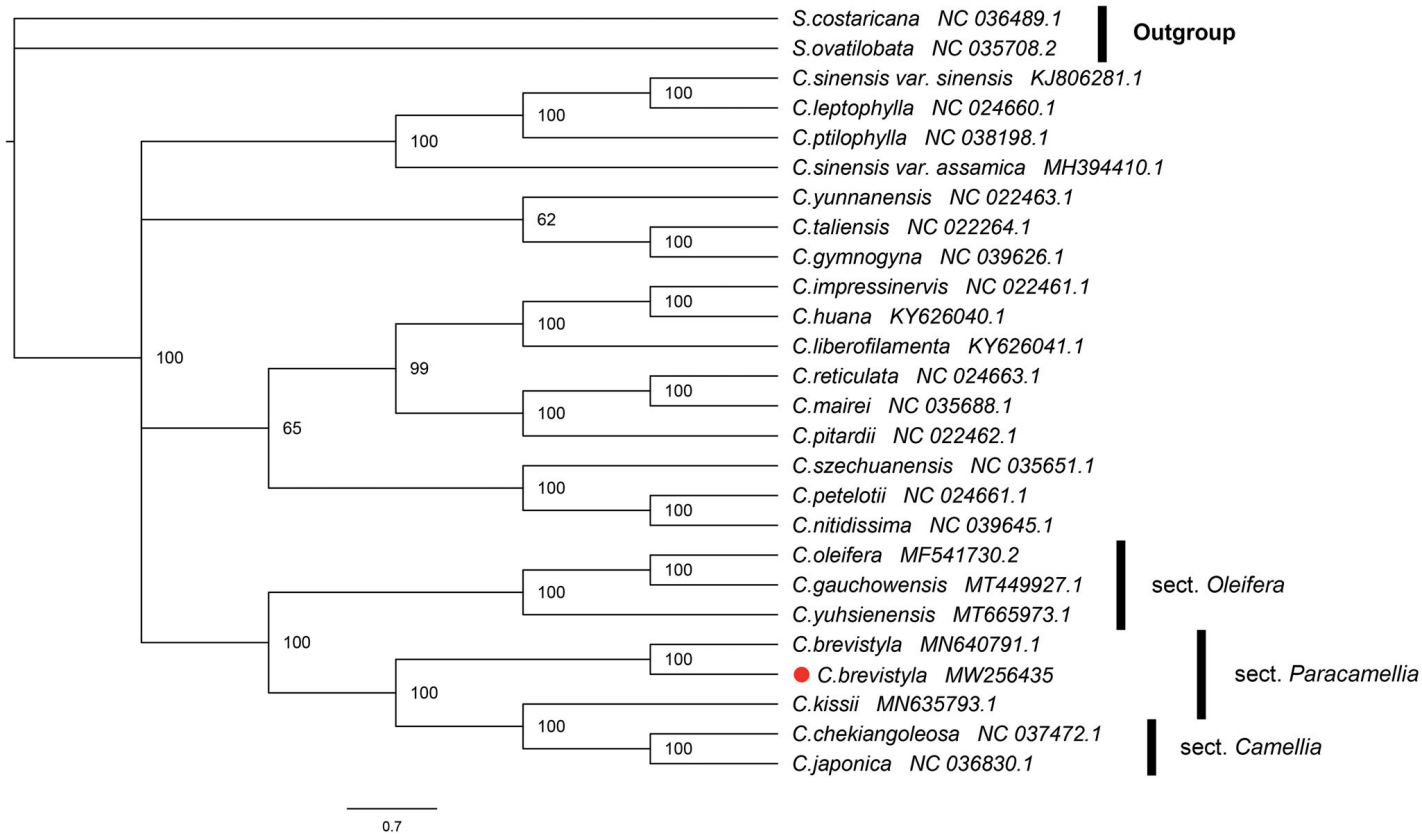
Bayesian-inference (BI) tree inferred using whole chloroplast genome sequences from 24 *Camellia* species. Number near the nodes represent the posterior probabilities.

## Data Availability

The genome sequence data that support the findings of this study are openly available in Genbank of NCBI (https://www.ncbi.nlm.nih.gov/) under the accession no. MW256435. The associated Bioproject, SRA, and Biosample numbers are PRJNA704218, SRX10153405, and SAMN18035138 respectively.

## References

[CIT0001] Chang HT. 1981. A taxonomy of genus *Camellia*. Guangzhou (China): The Editorial Staff of the Journal of Sun Yatsen University.

[CIT0002] Chang HT, Ren SX. 1998. Theaceae. In: Flora reipublicae popularis sinicae. Beijing (China): Science Press.

[CIT0003] Chiang SS, Hsu F-L, Hsu CK, Liu CF, Chu C. 2019. Role of *Camellia brevistyla* (Hayata) Coh. Stuart seed pomace extract on hypertension and vascular function in L‐NAME–treated mice. J Food Sci. 84(12):3555–3564. 31721202 10.1111/1750-3841.14913

[CIT0004] Huelsenbeck JP, Ronquist F. 2001. MRBAYES: Bayesian inference of phylogenetic trees. Bioinformatics. 17(8):754–755. 11524383 10.1093/bioinformatics/17.8.754

[CIT0005] Katoh K, Standley DM. 2013. MAFFT multiple sequence alignment software version 7: improvements in performance and usability. Mol Biol Evol. 30(4):772–780. 23329690 10.1093/molbev/mst010 PMC3603318

[CIT0006] Ming TL. 2000. Monography of the genus Camellia. Kunming (China): Yunnan Science and Technology Press.

[CIT0007] Ming TL, Zhang WJ. 1996. The evolution and distribution of genus *Camellia*. Acta Botanica Yunnanica. 18:1–13.

[CIT0008] Wang RY, Tung YT, Chen SY, Lee YL, Yen GC. 2019. Protective effects of camellia oil (*Camellia brevistyla*) against indomethacin-induced gastrointestinal mucosal damage in vitro and in vivo. J Funct Foods. 62:103539.

[CIT0009] Wang Y, Li J, Fan Z, Wu D, Yin H, Li X. 2020. Characterization of the complete chloroplast genome of *Camellia brevistyla*, an oil-rich and evergreen shrub. Mitochondrial DNA B Resour. 5(1):386–387. 33366568 10.1080/23802359.2019.1703607 PMC7748852

[CIT0010] Wu C-C, Tung Y-T, Chen S-Y, Lee W-T, Lin H-T, Yen G-C. 2020. Anti-inflammatory, antioxidant, and microbiota-modulating effects of camellia oil from *Camellia brevistyla* on acetic acid-induced colitis in rats. Antioxidants. 9(1):58. 31936300 10.3390/antiox9010058 PMC7022941

[CIT0011] Yang JB, Yang SX, Li HT, Yang J, Li DZ. 2013. Comparative chloroplast genomes of *Camellia* species. PloS One. 8(8):e73053. 24009730 10.1371/journal.pone.0073053 PMC3751842

[CIT0012] Zhang W, Kan SL, Zhao H, Li ZY, Wang XQ. 2014. Molecular phylogeny of Tribe Theeae (Theaceae s.s.) and its implications for generic delimitation. Plos One. 9(5):e98133. 24848365 10.1371/journal.pone.0098133 PMC4029964

